# Safety profile of 40% Garcinol from *Garcinia indica* in experimental rodents

**DOI:** 10.1016/j.toxrep.2018.06.009

**Published:** 2018-06-19

**Authors:** Muhammed Majeed, Sarang Bani, Beena Bhat, Anjali Pandey, Lakshmi Mundkur, Prakriti Neupane

**Affiliations:** a Sami Labs Limited, Bangalore, Karnataka, India; b Biological Research Department, Sami Labs Limited, Bangalore, Karnataka, India; c Phytochemical Research Department, Sami Labs Limited, Bangalore, Karnataka, India; d ClinWorld Private Limited, Bangalore, Karnataka, India

**Keywords:** Garcinol, Animal toxicity, OECD guidelines

## Abstract

The present study was taken up to evaluate the single dose acute toxicity, 28 days and 90 days repeated dose toxicity and reproductive/developmental toxicity of standardized 40% Garcinol in experimental rodents. The studies were conducted in compliance with OECD principles of good laboratory practice, guidelines for testing of chemicals no.420, 407, 408 and 421 respectively. Single dose acute oral toxicity was conducted on female Wistar rats as sighting study step-I (300 mg/kg) & sighting study step-II (2000 mg/kg) and main study (2000 mg/kg). Sub-acute, sub-chronic and reproductive/developmental studies were conducted in Wistar rats divided equally in vehicle control, 20, 50 and 100 mg/kg dose group along with recovery groups for vehicle control and high dose. Reproductive/developmental study was carried out for minimum of 28 days and in females during pregnancy and 4 days post partum. There were no abnormal clinical signs/behavioural changes, reproductive and developmental parameters, gross and histopathological changes as well as no alteration in the body weight, body temperature, haematology and other biochemical parameters in all the four studies. 40% Garcinol has a low toxicity profile in rodents and had no observed effects under experimental conditions used.

## Highlights

• Evaluated standardized 40% Garcinol in Wistar rats for its safety.
• Acute, sub-acute sub-chronic and reproductive/developmental toxicity study conducted.
• 40% Garcinol safe up to 2000 mg/kg at single dose.
• NOAEL of 40% Garcinol at sub acute, sub-chronic and reproductive/developmental study is 100 mg/kg/day.

## 1 Introduction

Food and health are the two terms always associated with each other through ages and today life sciences contribute to explain it as characterized by complexity [1]. Also, benefit of food beyond the basic nutritional value in prevention of disease and health enhancement using its elements is the interest of people today. Most of the bioactive components related to food products used daily, originated in plant are owed by the traditional medicinal practice [2]. There is continuous recognition of unique structured low molecular weight primary compounds from the natural components when compared to the combinatorial chemistry. Only the 10% of biodiversity has been analysed for their biological activity and numerous are there to be evaluated [3].

Likewise, *Garcinia indica* from Clusiaceae [4] family commonly known as Kokum is an evergreen plant native to Western Ghats of India but distributed throughout the tropical regions of India, Africa and China [5]. Kokum rind has been used as the alternative for tamarind or lemon in the culinary use and its extract in the beverages, gourmet spice and as carminative [6]. Ayurvedic system of medicine recommends its use in scurvy, rheumatism, oedema, sore, heat strokes, dermatological conditions, contagious diseases and as emollient and demulcent [7].

Polyisoprenylated benzophenone derivatives such as Garcinol and Isogarcinol, along with hydroxycitric acid, hydroxycitric acid lactone, citric acid and oxalic acid are predominantly found in *Garcinia indica* fruit rind [7]. The structural complexity and comprehensive biological activities draws the great attention to polyisoprenylated benzophenone class [4]. Thus, it is essential to have the safety profile of these derivatives. Garcinol like other polyisoprenylated benzophenones has the 1, 3- diketone system conjugated to the 3, 4- dihydroxybenzoyl moiety [8]. The various techniques have revealed that kokum’s antioxidant property is due to Garcinol and anthocyanins [9]. Garcinol’s oxidation sites C-3 ketonic group and hydroxyl group produces biologically active metabolites [7]. Cell signalling pathways associated with apoptosis and tumour growth have been found to be regulated by Garcinol [10]. Garcinol has been studied for its anticancer [[11], [12], [13], [14], [15], [16], [17]], antimicrobial property [18], anti-diabetic [19] and anti-inflammatory properties [20] and also its use in seizure management [21].

Sami labs limited has standardized 40% Garcinol preparation for its efficacy studies at the doses not exceeding 10 mg/kg with significant outcomes (data not shown). It has various health benefits; progression of Garcinol into a pharmacologically active molecule needs further toxicological and pharmacokinetic studies which are required to establish the safety [20].

The present work was taken up to summarise acute, sub-acute, sub-chronic and reproductive/developmental toxicity studies of 40% Garcinol in the experimental animals.

## 2 Materials and method

### 2.1 Chemicals

The AR (Analytical Grade) solvents and chemicals were used in all toxicity studies. The 40% Garcinol i.e. test sample with batch no. C120775 was prepared by phytochemical group at Sami Labs Limited. The reference sample (C120775) has been stored at Sami Labs Limited.

**Fig. 1:**
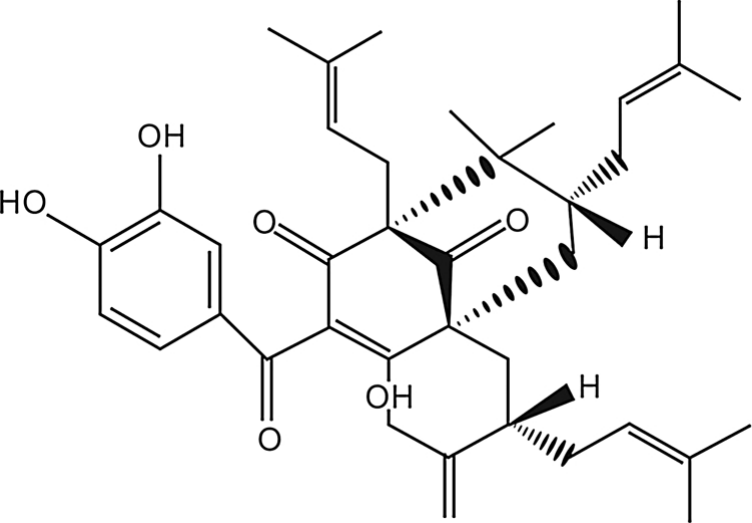
Garcinol.

**Fig. 2:**
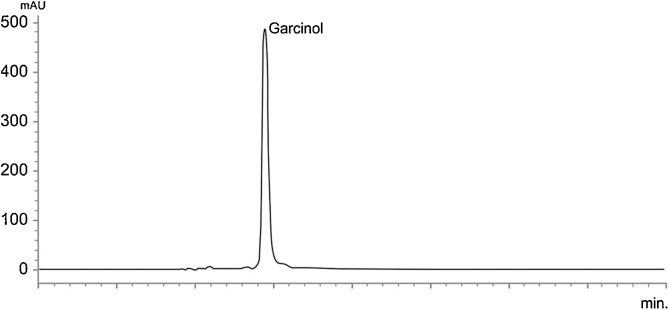
HPLC Assay of 40% Garcinol.

Garcinol (Crystallized yellow needles) chemically polyisoprenylated benzophenone derivative of molecular formula C_38_H_50_O_6_ (Fig. 1) with molecular weight of 602.39252 and melting point of 122 °C is obtained from *Garcinia indica* fruit rind [5,20]. Dried *Garcinia indica* fruit rind was extracted with hexane and fractionated by column chromatography. Hexane extract was completely crystallized in ethanol and vacuum dried. Thus, obtained residue of Garcinol was dissolved in cold hexane and re-crystallized at room temperature to obtain as pale yellow needle like crystals [10]. The acquired Garcinol from the above process was standardized to 40% with microcrystalline cellulose powder. The HPLC graph of standardized sample at 240 nm is as shown (Fig. 2).

### 2.2 Animals

Wistar Rats of 6–10 weeks old, housed maximum of 5 per sex per cage in a polypropylene cage with maintained room temperature of 20.2–23.5 °C, relative humidity of 30–70% with 12 h fluorescent light and 12 h dark cycle were used in all the toxicity studies. Animals were fed with Nutrilab rodent feed (Provimi Animal nutrition Pvt. Ltd) and purified water (aqua guard water filter) that was provided ad libitum. Animals were kept in sterilized rice husk beddings changed along with the cage twice a week during acclimatization and entire experimental study period.

### 2.3 Ethics

Each separate protocol for each (acute, sub acute, sub chronic and reproductive/developmental toxicity) study was approved by Institutional Animal Ethics Committee of CSIR-Indian Institute of Toxicology Research, Lucknow (registration no. 54/99/CPCSEA) and of Bioneeds, Bangalore (registration no. 969/bc/06/CPCSEA) respectively. The studies were conducted in accordance with the recommendation of the committee for the purpose of control and supervision of experiments on animals (CPCSEA) guidelines for laboratory animal facility published in the gazette of India, December 15th 1998. Also the studies were in compliance with OECD principles of good laboratory practice, guidelines for testing of chemicals no.420, 407, 408 and 421 and in accordance with SOP of the institution.

### 2.4 Acute oral toxicity study

Required quantity of 40% Garcinol was weighed and then mixed with 0.5% w/v Carboxy methyl cellulose. Volume was made up with 0.5% w/v Carboxy methyl cellulose in the measuring cylinder in accordance with the required dose. The 40% Garcinol was administered orally by gavage as single dose using gavage needle number 14. Dose was adjusted according to the body weight which was measured on the dosing day and dose volume was 10 ml/kg. Food was offered 3–4 hrs after dosing. The dose given was 300 mg/kg body weight, 2000 mg/kg body weight and 2000 mg/kg body weight in sighting study step–I, sighting study step–II and main study respectively. All the animals were observed for clinical signs of toxicity and mortality at 30–40 min, 1 h ( ± 10 min), 2 h ( ± 10 min), 3 h ( ± 10 min) and 4 h ( ± 10 min) on the day one after dosing and thereafter once daily for clinical signs and twice daily for mortality during the 14 days study period. All the test animals were weighed on day 1 before the dose administration and on day 7 and 14 during the study period. All the animals were sacrificed at term by overdose of CO2 and subjected to necroscopy.

### 2.5 Sub acute or 28 days repeated dose toxicity study

The formulation of 40% Garcinol was prepared fresh daily and vehicle was administered at the dose volume of 10 mg/kg body weight. The animals were grouped 20, 50 and 100 mg/kg/day (Table 1) as there was no statistically significant toxicity in single dose toxicity and not more than 5–10 mg/kg dose showed effect in the efficacy study (data not shown). The dose volume was calculated for individual animals according to body weight during the treatment period. Dose administration was done by oral gavage once daily for four weeks. There was no administration of 40% Garcinol formulation/vehicle during the 14 days recovery period.

**Table 1:** Dose grouping of sub acute toxicity study.

Group no	Treatment groups	Dose (mg/kg/day)	Concentration (mg/ml)	No. Of rats	Sex
G1	Vehicle control	0	NA	5	M
				5	F
G2	Low dose	20	2	5	M
				5	F
G3	Mid dose	50	5	5	M
				5	F
G4	High dose	100	10	5	M
				5	F
^*^G1R	Vehicle control	0	NA	5	M
				5	F
^*^G4R	High dose	100	10	5	M
				5	F

M-male, F-female, NA- not available, ^*^ Recovery groups with the 14 days recovery period.

Rats were observed for clinical changes and were recorded prior to the dose formulation and weekly during the study period. Changes in skin and fur, eyes, mucous membrane, occurrence of secretions and excretions, changes in gait, posture and presence of any change in general behaviour of animals were observed. Individual body weights were recorded before dosing (day 1) and weekly thereafter. Fasting body weights were recorded before sacrifice. Body weights were recorded from recovery animals after cessation of dosing, during the recovery period. Fasted body weights were used for calculation of organ: body weight ratio. The food consumption was measured as average cage-wise food intake (g/rat/day) at weekly intervals during in-life phase of the experiment.

The collection of blood sample at the end of study period (28^th^ day for main groups & 42^nd^ day for recovery groups) was done into K_2_ EDTA tubes for haematology and tubes without anticoagulant for clinical chemistry. Haematological parameters were determined using Sysmex XT 1800 IV haematology analyzer and biochemistry parameters analyzed using Rx Daytona (Randox) automatic analyzer after serum was separated in a refrigerated centrifuge at approximately 5000 rpm for 10 min. The finding from detailed necropsy of all group rats was recorded and the animals were examined visually for external abnormalities including palpable masses. Tissues and organs collected on completion of the gross pathology examination from all animals were preserved in 10% formaldehyde.

### 2.6 Sub chronic or 90 days repeated dose toxicity

The dose grouping of the animals is as shown in Table 2. The particular doses were taken with reference to the 28 days toxicity study as there was no dose related toxicity so to observe its effect in long term. The individual animal dose was determined by the individual weight taken before dosing and weekly thereafter during the study period. The 40% Garcinol or vehicle formulation was administered by oral gavage to all the groups once daily at approximately the same time each day for a period of thirteen weeks. There was no administration of 40% Garcinol formulation/vehicle during the 14 days recovery period.

**Table 2:** Dose grouping of sub chronic toxicity study.

Group no	Treatment groups	Dose (mg/kg/day)	Concentration (mg/ml)	No. Of rats	Sex
G1	Vehicle control	0	NA	10	M
				10	F
G2	Low dose	20	2	10	M
				10	F
G3	Mid dose	50	5	10	M
				10	F
G4	High dose	100	10	10	M
				10	F
G1R^*^	Vehicle control	0	NA	5	M
				5	F
G4R^*^	High dose	100	10	5	M
				5	F

M-male, F-female, NA- not available, ^*^ Recovery groups with 14 days recovery period.

Rats were observed for clinical changes and were recorded prior to the dosing and weekly during the study period. Changes in skin and fur, eyes, mucous membrane, occurrence of secretions and excretions, changes in gait, posture and presence of any change in general behaviour of animals were observed. Fasting body weights were recorded before sacrifice. Body weights were recorded from recovery animals after cessation of dosing, during the recovery period. Fasted body weights were used for calculation of organ: body weight ratio. The food consumption was measured as average cage-wise food intake (g/rat/day) at weekly intervals during in-life phase of the experiment.

The collection of blood sample at the end of study period (90 days for main groups and 104 days for recovery groups) was done into K_2_ EDTA tubes for haematology and tubes without anticoagulant for clinical chemistry. Haematological parameters were determined using Sysmex XT 1800 IV haematology analyzer and biochemistry parameters analyzed using Rx Daytona (Randox) automatic analyzer after serum was separated in a refrigerated centrifuge at approximately 5000 rpm for 10 min. The finding from detailed necropsy of all group rats was recorded and the animals were examined visually for external abnormalities including palpable masses. Tissues and organs collected on completion of the gross pathology examination from all animals were preserved in 10% formaldehyde.

### 2.7 Reproduction/developmental toxicity study

The vehicle used to make up the volume of 40% Garcinol was Carboxy methyl cellulose and dose grouping was done as shown in Table 3. The dose formulations and vehicle was administered according to body weight everyday for a period of 14 days prior to mating, during mating and in females during mating, pregnancy and day 4 of post partum of pups. One male and one female rat were mated (1:1), the female was placed with same male until pregnancy occurs or two weeks were elapsed. Each morning females were examined for the presence of sperm or vaginal plugs. Day 0 of pregnancy is considered as the day a vaginal plug or sperm were found. In case pairing is unsuccessful, remating of females with proven males of same group was considered.

**Table 3:** Dose Grouping for reproductive and developmental study.

Group no	Treatment groups	Dose (mg/kg/day)	No. Of rats	Sex
G1	Vehicle control	0	10	M
			10	F
G2	Low dose	20	10	M
			10	F
G3	Mid dose	50	10	M
			10	F
G4	High dose	100	10	M
			10	F

M-male, F-female.

Rats were observed for changes as in sub chronic study for the presence of abnormal behaviour, if any. Body weights of the animals were taken on day 1 of dosing and weekly thereafter. During gestation period, females were weighed on days 0, 7, 14 and 20, after parturition and day 4 postpartum. Pups at birth and day four were counted and weighed individually. All the surviving animals were sacrificed and were examined microscopically. The animals were examined visually for external abnormalities and microscopic examination of reproductive organs. The counting of implantation sites and number of corpora lutea was done. The tissues were processed for routine paraffin embedding and sections were stained with haematoxyline and Eosin statin. Histopathological examination of low dose and mid dose was not carried out as preserved organs of vehicle control and high dose group rats didn’t show gross histopathological changes.

## 3 Statistical analysis

The data generated was subjected to statistical evaluation for the significance of any 40% Garcinol induced changes and their interpretation of potential for toxicity. The statistical analysis of the experimental data was carried out by the one-way ANOVA test. Statistical comparisons were evaluated at the 5% (P ≤ 0.05) significance level. For reproductive/developmental toxicity studies, all quantitative variables like laboratory investigations (pups size and pups survival ratio) were subjected to Fishers Student T-test and Wilcoxon Rank Sum test. In the case of recovery groups also, data was analyzed using the methods stated above. Comparison of means between treatment groups was performed.

## 4 Results

In acute oral toxicity study with the single high dose of 2000 mg/kg body weight didn’t show any significant clinical signs and mortality in the female Wistar rats. The body weight gain was comparable during study period as shown in Table 4.

**Table 4:** Effect of single dose oral exposure to 40% Garcinol on Body weight and body gain percent in Wistar rats.

Study Type	Dose (mg/kg)	No. of animals	Sex		Body weight on days			Body weight gain %	
					1	7	14	1-7	7-14
Slighting study– Step-I	300	1	Female	l 151.32		174.25	186.35	15.16	23.15
Slighting study - step-II	2000	1	Female	l 165.88		181.69	197.24	9.53	18.91
Main study	2000	4	Female	Mean 161.41 SD ± 3.13		174.47 ± 3.73	193.51 ± 5.35	8.09 ± 1.02	18.01 ± 1.38

NAD-no abnormality observed.

**Fig. 3:**
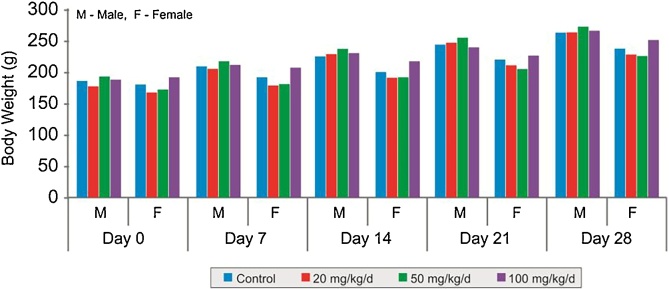
Comparison of mean body weights of Wistar rats in different dose groups in the 28 days repeated dose toxicity study of 40% Garcinol.

In 28 days repeated dose toxicity study, there were no clinical signs of toxicity and mortality noticed in any of the treatment or control group animals. There were no differences in the body weight (Fig. 3), body temperature and food consumption changes between test and control group during the course of study and recovery period. The haematological parameters (Table 5), biochemical parameters (Table 6), pathological examination and organ weight (Table 7) at necropsy showed no significant changes.

**Table 5:** Effect of 28 days exposure to 40% Garcinol on haematological parameters in Wistar rats.

Parameter	Sex	Control	Control recovery	Treated			
				20 mg/kg/d	50 mg/kg/d	100 mg/kg/d	100 mg/kg/d recovery
WBC (10^3^cells/μl)	M	13.32	14.95	11.70	6.70	13.64	13.73
	F	6.04	10.34	7.65	6.84	7.88	7.70
RBC (10^6^cells/μl)	M	7.59	8.80	8.55	7.66	8.81	8.99
	F	8.13	7.98	7.79	8.13	8.13	9.06
Haemoglobin (g/dl)	M	13.14	14.46	14.52	13.38	14.80	14.66
	F	14.10	13.94	13.46	14.02	13.86	14.62
Haematocrit (%)	M	41.76	43.80	44.04	41.60	46.32	44.30
	F	42.60	41.64	42.34	42.52	42.20	43.30
MCV (fL)	M	55.12	49.80	51.58	54.44	52.70	49.34
	F	52.66	52.16	54.54	52.40	51.94	47.80
MCH (pg)	M	17.36	16.46	17.00	17.48	16.84	16.34
	F	17.42	17.46	17.28	17.26	17.06	16.14
MCHC (g/dl)	M	31.60	33.02	32.98	32.16	31.94	33.08
	F	33.08	33.50	31.80	32.98	32.84	33.76
Platelet (10^3^cells/μl)	M	1151.20	978.40	1343.80	1167	978.40	1179.20
	F	1144.60	1046	1206.80	1258.60	1285	1093.60
Neutrophils (%)	M	11.88	1.54	14.60	16.94	16.44	1.81
	F	19.10	1.32	18.46	18.68	19.22	1.42
Lymphocytes (%)	M	80.14	12.25	76.10	74.24	75.20	10.95
	F	66.92	8.12	71.40	69.56	70.36	5.49
Monocytes (%)	M	6.90	0.62	8.08	7.26	6.04	0.65
	F	8.38	0.50	7.20	6.46	5.60	0.46
Eosinophils (%)	M	0.90	0.39	1.04	1.22	2.16	0.25
	F	5.36	0.39	2.84	5.18	4.72	0.32
Basophiles (%)	M	0.18	0.03	0.18	0.24	0.16	0.02
	F	0.24	0.01	0.10	0.12	0.10	0.01

Values are in mean, M-Male, F-Female, n = 5.

**Table 6:** Effect of 28 days exposure to 40% Garcinol on biochemical parameters in Wistar rats.

Parameter	Sex	Control	Treated			Control recovery	100 mg/kg recovery
			20 mg/kg	50 mg/kg	100 mg/kg		
Glucose (mg/dl)	M	77	68.89	63.86	75.30	77.72	90.79
	F	77.59	74.02	70.44	73.06	84.64	101.95
Creatinine (mg/dl)	M	0.54	0.52	0.50	0.48	0.72	0.71
	F	0.61	0.56	0.52	0.54	1.21	0.75
AST (U/L)	M	249.50	224.33	190	172.8	167	128.60
	F	304.33	278	178.50	222.50	130.61	134.20
Urea (mg/dl)	M	39.56	35.07	31.54	33.88	44.08	42.56
	F	48.99	37.91	36.48	34.71	46.34	43.49
Total protein (mg/dl)	M	6.16	6.46	6.14	6.30	6.70	6.98
	F	6.62	6.74	6.80	6.96	6.32	7.46
Triglycerides (mg/dl)	M	132.80	126.32	145.81	98.69	130.29	136.26
	F	99.23	77.62	128.75	94.38	89.24	109.78
Albumin (mg/dl)	M	3.16	3.08	3.14	3.14	3.06	3.19
	F	3.24	3.33	3.34	3.47	24.76	3.46
Uric acid (mg/dl)	M	1.00	1.05	1.09	1.04	1.01	0.91
	F	1.13	1.04	0.93	0.95	0.86	0.92
Cholesterol (mg/dl)	M	53.52	61.78	50.75	56.50	63.90	67.46
	F	55.75	50.28	65.67	51.99	70.85	64.06
Globulin (mg/dl)	M	3.00	3.38	3.00	3.18	3.64	3.79
	F	3.38	3.41	3.46	3.49	3.95	4.00
ALT (U/L)	M	65.20	72.60	54.80	52.80	62.40	52.60
	F	66.40	57.60	52	51.20	47	55.40
ALP (U/L)	M	173.80	228.40	243.40	210.60	0.16	0.18
	F	151.40	106.40	117.80	128.40	0.15	0.20
Na (mmol/L)	M	145.67	135.67	123.67	113	133.67	115.33
	F	153.33	137.33	120.33	127.33	118.3	122
K (mg/dl)	M	4.86	4.96	4.08	4.10	4.62	4.01
	F	5.34	4.89	4.15	4.33	4.23	4.26
Cl (mg/dl)	M	98.33	91	85	77	90.33	77.33
	F	101.67	93.67	82.67	86	81	84.33

Values are in mean, M-Male, F-Female, n = 5.

**Table 7:** Effect of 28 days exposure to 40% Garcinol on mean organ weights(g) in Wistar Rats.

Organs	Sex	Control	Treated			Control recovery	100 mg/kg recovery
			20 mg/kg	50 mg/kg	100 mg/kg		
Liver	M	10.10	9.75	10.71	8.65	10.53	9.58
	F	8.03	6.75	6.81	7.29	7.57	7.04
Kidney	M	2.30	2.21	2.28	1.96	2.35	2.21
	F	1.50	1.46	1.44	1.57	1.80	1.56
Heart	M	1.16	1.17	1.12	0.95	1.15	1.21
	F	0.82	0.77	0.85	0.99	0.93	0.85
Spleen	M	1.27	0.87	1.22	1.24	1.18	1.18
	F	0.80	0.85	0.77	0.90	0.84	0.75
Lung	M	2.69	2.52	2.36	2.44	2.45	2.33
	F	1.99	1.74	1.90	1.73	1.89	1.98
Adrenal	M	0.06	0.07	0.04	0.05	0.06	0.05
	F	0.07	0.08	0.05	0.08	0.07	0.05
Brain	M	1.87	1.78	1.76	1.86	2.00	1.82
	F	1.73	1.73	1.70	1.73	1.89	1.98
Testes	M	3.06	3.01	3.14	2.76	3.05	3.13
Ovaries	F	0.17	0.13	0.13	0.91	0.16	0.13
SV BU Prostate	M	1.86	1.64	1.95	1.66	2.05	2.12
Uterus	F	0.51	0.60	0.61	0.62	0.71	0.66
Epididymus	M	1.14	1.19	1.27	0.97	1.16	1.35

M-Male, F-Female, n = 5.

**Fig. 4:**
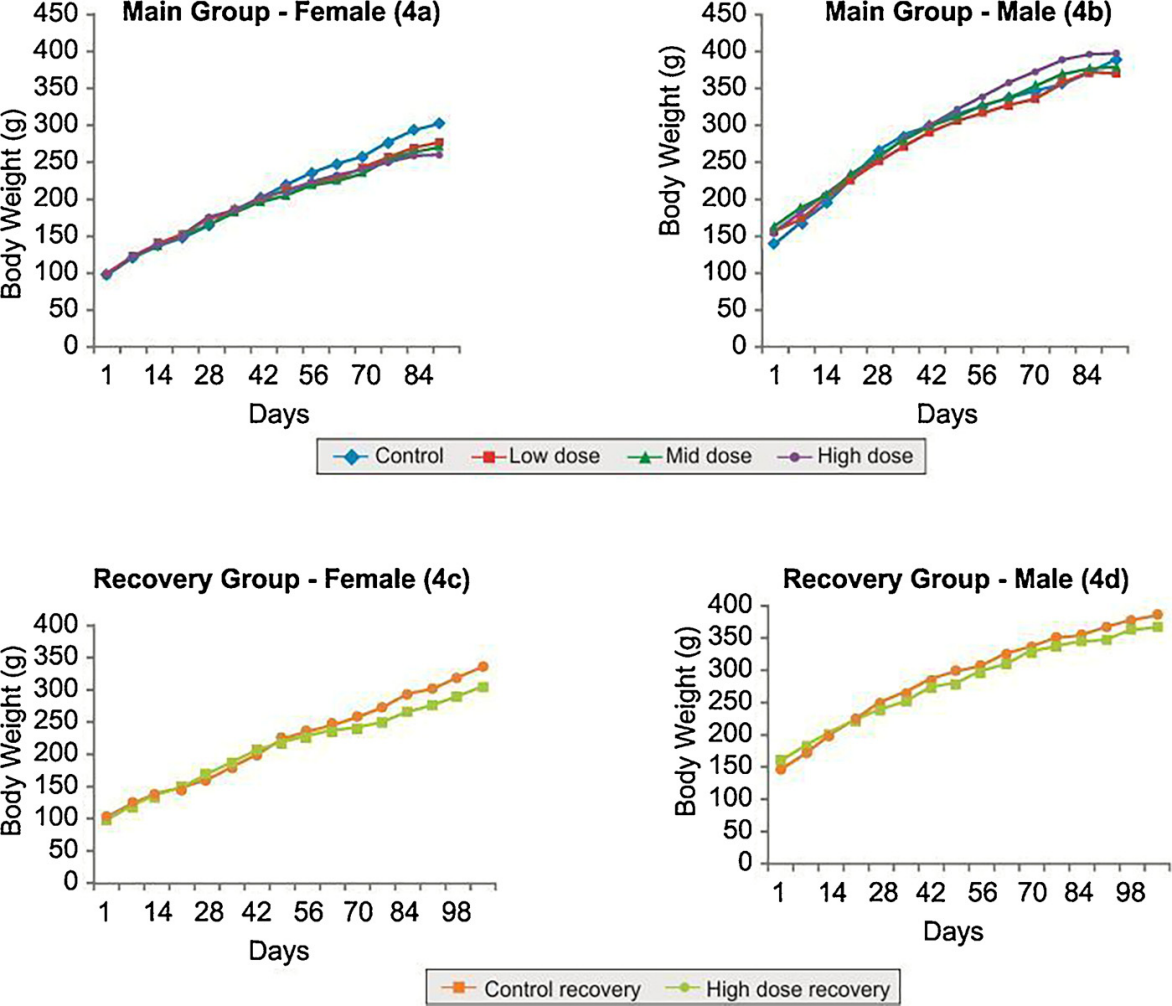
Line plots showing body weights of animals in 90 days repeated dose toxicity study of 40% Garcinol.

In 90 days repeated dose toxicity study, there were no abnormal clinical signs and mortality in any of the treatment or control group animals. There was no significant change in body weight gain over the study period (Fig. 4). The haematological parameters (Table 8), biochemical parameters (Table 9), absolute and relative organ weight (Table 10) of the animals are as shown.

**Table 8:** Effect of 90 days exposure to 40% Garcinol on haematological parameters in Wistar rats.

Parameter	Sex	Control	Treated			Control recovery	100 mg/kg recovery
			20 mg/kg	50 mg/kg	100 mg/kg		
WBC (10^3^cells/μl)	M	13.77 ± 4.81	18.57 ± 5.28	16.08 ± 3.88	17.16 ± 7.77	8.10 ± 4.93	13.85 ± 7.46
	F	8.32 ± 1.84	8.91 ± 3.37	7.01 ± 2.57	9.19 ± 2.85	7.21 ± 4.10	5.62 ± 2.24
RBC (10^6^cells/μl)	M	9.02 ± 0.46	9.11 ± 0.66	8.96 ± 0.45	8.99 ± 0.56	9.22 ± 0.42	8.49 ± 0.68
	F	8.11 ± 0.47	8.02 ± 0.31	8.31 ± 0.41	8.31 ± 0.39	7.89 ± 0.45	8.02 ± 0.26
Haemoglobin (g/dl)	M	13.95 ± 0.54	14.39 ± 0.92	14.23 ± 0.45	14.63 ± 0.80	14.66 ± 0.44	13.78 ± 0.48
	F	13.62 ± 0.73	13.75 ± 0.41	14.20 ± 0.54	14.10 ± 0.72	13.08 ± 0.71	13.54 ± 0.50
Haematocrit (%)	M	40.81 ± 1.12	42.39 ± 2.53	41.22 ± 1.18	42.41 ± 2.12	41.96 ± 1.07	39.88 ± 1.34
	F	41.05 ± 2.52	40.76 ± 1.45	41.03 ± 1.40	39.32 ± 7.82	38.35 ± 1.65	69.94 ± 1.22
MCV (fL)	M	45.31 ± 1.63	46.59 ± 1.46	46.02 ± 1.96	48.36 ± 4.19	45.58 ± 1.69	47.20 ± 3.81
	F	50.65 ± 1.48	50.86 ± 1.44	49.40 ± 1.72	48.90 ± 5.71	48.63 ± 1.19	49.82 ± 1.58
MCH (pg)	M	15.55 ± 0.62	15.81 ± 0.56	15.89 ± 0.59	38.52 ± 44.04	15.92 ± 0.58	16.30 ± 1.22
	F	16.79 ± 0.46	17.14 ± 0.45	17.09 ± 0.45	17.02 ± 0.74	16.60 ± 0.29	16.88 ± 0.60
MCHC (g/dl)	M	34.37 ± 0.53	33.93 ± 0.44	37.81 ± 10.55	34.50 ± 0.42	34.94 ± 0.57	34.56 ± 0.23
	F	33.17 ± 0.47	33.74 ± 0.36	34.60^*^±0.44	33.49 ± 0.41	34.10 ± 0.47	27.88 ± 13.69
Platelet (10^3^cells/μl)	M	877 ± 100.49	972.10 ± 126.24	943 ± 57.58	878 ± 105.53	836.80 ± 142.48	930.80 ± 148.06
	F	929.80 ± 69.69	794.10 ± 279.39	873.50 ± 107.48	896.90 ± 113.59	761.25 ± 80.66	899.6 ± 157.60
Neutrophils (%)	M	15.30 ± 5.31	16.47 ± 9.38	17.06 ± 3.68	17.71 ± 8.62	18.66 ± 9.32	10.00 ± 3.00
	F	21.40 ± 5.30	21.51 ± 10.99	20.24 ± 5.44	18.42 ± 3.36	12.63 ± 7.94	18.38 ± 2.65
Lymphocytes (%)	M	76.39 ± 5.74	75.00 ± 10.69	74.53 ± 3.87	74.12 ± 9.87	69.90 ± 20.64	82.60 ± 3.71
	F	66.83 ± 8.04	71.05 ± 7.97	69.16 ± 8.45	71.19 ± 4.68	80.28 ± 9.90	73.80 ± 3.89
Monocytes (%)	M	4.73 ± 1.45	4.83 ± 1.14	4.44 ± 1.16	5.12 ± 1.52	2.68 ± 0.97	3.56 ± 0.94
	F	4.54 ± 1.42	6.13 ± 1.73	5.44 ± 1.68	4.76 ± 0.82	4.15 ± 2.10	4.00 ± 1.09
Eosinophils (%)	M	3.38 ± 1.76	3.48 ± 1.46	3.85 ± 1.30	2.89 ± 2.34	2.58 ± 0.75	3.72 ± 1.03
	F	7.09 ± 3.78	4.48 ± 2.09	5.08 ± 3.84	5.52 ± 1.47	2.75 ± 2.09	3.60 ± 2.31
Basophils (%)	M	0.16 ± 0.11	0.16 ± 0.05	0.12 ± 0.04	0.14 ± 0.09	0.20 ± 0.29	0.12 ± 0.04
	F	0.11 ± 0.06	0.14 ± 0.10	0.13 ± 0.10	0.14 ± 0.05	0.20 ± 0.16	0.22 ± 0.24

Values are mean ± SD, M-Male, F-Female, n = 10, P values: ^*^ <0.05 (values of low, mid and high dose group were compared with control).

**Table 9:** Effect of 90 days exposure to 40% Garcinol on biochemical parameters in Wistar Rats.

Parameter	Sex	Control	Treated			Control recovery	100 mg/kg recovery
			20 mg/kg	50 mg/kg	100 mg/kg		
Glucose mg/dl)	M	72.15 ± 19.21	77.76 ± 24.95	65.35 ± 18.01	83.87 ± 16.43	87.61 ± 16.23	94.55 ± 2.81
	F	82.07 ± 27.87	67.16 ± 29.64	73.50 ± 26.44	148.43^*^±70.46	95.25 ± 8.42 ±	121.70^*^±21.55
Creatinine (mg/dl)	M	0.56 ± 0.07	0.62 ± 0.07	0.41^*^ ±0.12	0.59 ± 0.07	0.53 ± 0.10	0.60 ± 0.05
	F	0.47 ± 0.12	0.40 ± 0.14	0.54 ± 0.03	0.59^*^±0.08	0.62^*^±0.04	0.61^*^±0.07
AST (U/L)	M	144.86 ± 44.72	209.90 ± 34.44	111.60 ± 40.25	139.60 ± 28.65	139.0 ± 26.42	148.80 ± 19.45
	F	129 ± 52.38	102.00 ± 51.59	192.89^*^±59.17	107.10 ± 25.64	133.75 ± 21.62	127.80 ± 32.48
Urea (mg/dl)	M	36.69 ± 4.46	39.21 ± 4.79	28.65^*^±7.61	36.92 ± 4.39	36.57 ± 4.24	39.05 ± 3.11
	F	24.26 ± 5.70	23.49 ± 8.37	37.80^*^±5.55	28.05 ± 4.91	37.96^*^±0.68	30.66 ± 4.13
Total protein(mg/dl)	M	6.30 ± 0.83	6.85 ± 0.95	4.60^*^±1.27	6.74 ± 0.48	7.18 ± 0.90	7.42 ± 0.63
	F	5.33 ± 1.62	4.51 ± 1.75	7.63 ± 0.35	7.24 ± 0.75	7.95 ± 0.30	7.82 ± 0.86
Triglycerides (mg/dl)	M	86.95 ± 22.87	108.51 ± 73.28	53.76^*^±35.23	111.65 ± 26.07	96.03 ± 65.67	103.97 ± 36.04
	F	64.54 ± 24.11	112.81 ± 61.61	87.03^*^±37.19	99.44^*^±61.12	142.87^*^±10.18	80.72^*^±6.20
Albumin (mg/dl)	M	2.80 ± 0.19	3.52^*^±0.40	2.53 ± 0.70	2.99 ± 0.26	3.48 ± 0.30	3.14 ± 0.21
	F	3.22 ± 0.71	2.60^*^±0.82	4.21^*^ ±0.35	3.37^*^±0.21	3.60 ± 0.17	3.71 ± 0.52
ALP (U/L)	M	317.30 ± 84.09	390.90 ± 182.83	301.29 ± 153.71	243.00 ± 87.60	354.10 ± 208.28	566.74 ± 251.00
	F	106.80 ± 32.57	128.30 ± 41.99	374.11 ± 544.43	270.20 ± 380.76	446.15 ± 160.49	469.77 ± 274.45
Uric acid (mg/dl)	M	1.58 ± 0.82	2.35^*^±1.13	1.30 ± 0.65	1.44 ± 0.39	1.07 ± 0.32	1.01 ± 0.09
	F	1.33 ± 0.41	1.50 ± 1.03	0.88 ± 0.14	2.37^*^±1.64	1.14 ± 0.44	1.94 ± 0.61
Cholesterol (mg/dl)	M	63.20 ± 10.45	68.18 ± 15.50	56.20 ± 12.39	66.48 ± 10.81	65.14 ± 12.26	54.07 ± 12.36
	F	66.68 ± 19.67	57.45 ± 18.21	77.80 ± 14.14	72.56 ± 15.53	61.74 ± 6.11	63.97 ± 8.69
Globulin mg/dl)	M	3.60 ± 0.37	3.33 ± 0.71	2.07^*^±0.65	3.75 ± 0.38	3.70 ± 0.85	4.28 ± 0.49
	F	2.08 ± 1.03	1.91 ± 0.96	3.42 ± 0.37	3.87^*^±0.60	4.34^*^±0.33	4.11^*^±0.37
ALT (U/L)	M	51.60 ± 10.91	69.70^*^±12.22	46.80 ± 13.55	58.20 ± 9.16	56.60 ± 11.72	62.80 ± 4.66
	F	42.60 ± 16.45	35.67 ± 12.08	49.44 ± 10.81	42.60 ± 9.72	52.50 ± 5.44	40.00 ± 20.46
Total bilirubin (mg/dl)	M	0.08 ± 0.06	0.11 ± 0.10	0.09 ± 0.03	0.04 ± 0.07	0.10 ± 0.14	0.10 ± 0.07
	F	0.06 ± 0.05	0.06 ± 0.07	0.11 ± 0.03	0.04 ± 0.05	0.22 ± 0.17	0.08 ± 0.08

Values are mean ± SD, M-Male, F-Female, n = 10, P values: ^*^ <0.05 (values of low, mid and high dose group were compared with control).

**Table 10:** Effect of 90 days exposure to 40% Garcinol on relative organ weight in Wistar rats.

Organs	sex	Control	Treated			Control recovery	100 mg/kg recovery
			20 mg/kg	50 mg/kg	100 mg/kg		
Liver	M	2.96 ± 0.431	3.31 ± 0.772	3.02 ± 0.418	3.18 ± 0.326	3.18 ± 0.738	2.71 ± 0.302
	F	2.48 ± 0.378	2.68 ± 0.294	2.40 ± 0.472	2.90^*^ ±0.313	2.36 ± 0.341	2.44 ± 0.749
Kidney	M	0.73 ± 0.169	0.67 ± 0.142	0.72 ± 0.088	0.67 ± 0.107	0.74 ± 0.156	0.68 ± 0.104
	F	0.53 ± 0.056	0.59 ± 0.065	0.65 ± 0.094	0.87^*^ ±0.248	0.54 ± 0.08	0.61 ± 0.178
Heart	M	0.35 ± 0.109	0.32 ± 0.065	0.29 ± 0.046	0.33 ± 0.035	0.36 ± 0.088	0.30 ± 0.026
	F	0.31 ± 0.03	0.30 ± 0.038	0.40^*^±0.066	0.34 ± 0.055	0.28 ± 0.031	0.30 ± 0.097
Spleen	M	0.41 ± 0.173	0.30 ± 0.079	0.30 ± 0.044	0.27^*^ ±0.077	0.29 ± 0.058	0.28 ± 0.046
	F	0.26 ± 0.028	0.30 ± 0.057	0.30 ± 0.068	0.26 ± 0.088	0.25 ± 0.001	0.01 ± 0.004
Adrenal	M	0.01 ± 0.008	0.02 ± 0.004	0.01 ± 0.005	0.01 ± 0.006	0.02 ± 0.003	0.02 ± 0.003
	F	0.02 ± 0.004	0.02 ± 0.003	0.02 ± 0.003	0.02 ± 0.007	0.02 ± 0.001	0.01 ± 0.004
Brain	M	0.49 ± 0.084	0.50 ± 0.073	0.53^*^ ±0.06	0.41^*^ ±0.096	0.50 ± 0.11	0.53 ± 0.061
	F	0.63 ± 0.040	0.65 ± 0.097	0.78 ± 0.146	0.73 ± 0.077	0.56 ± 0.038	0.53 ± 0.159
Testes	M	0.81 ± 0.108	0.84 ± 0.129	0.84 ± 0.097	0.79^*^ ±0.118	0.97 ± 0.168	0.83 ± 0.082
Ovaries	F	0.29 ± 0.069	0.26 ± 0.160	0.24 ± 0.035	0.41 ± 0.126	0.28 ± 0.079	0.56 ± 0.295
Uterus	F	0.06 ± 0.007	0.04 ± 0.023	0.09^*^ ±0.016	0.09^*^ ±0.041	0.03 ± 0.005	0.17 ± 0.138
Epididymus	M	0.48 ± 0.165	0.34 ± 0.164	0.38 ± 0.119	0.35 ± 0.086	0.38 ± 0.073	0.30 ± 0.042

Values are mean ± SD, M-Male, F-Female, n = 10, P values: ^*^ <0.05 (values of low, mid and high dose group were compared with control).

In reproductive and developmental toxicity study, none of the animals in any groups, both control and test item treated exhibited any abnormal clinical signs/behavioural changes and mortality that may be attributed to the test item. There was no loss in body weight or difference in body weight (Table 11, Table 12) as well as organ weight (Table 13) between control and treated groups during the study period. The reproductive/developmental parameters of the animals were comparable as shown in the Table 14.

**Table 11:** Body weight of males in reproductive/developmental study.

Treatment (mg/kg/d)	Day 0	Day 7	Day 14	Day 21	Day 28
Control	184 ± 19.37	206 ± 22.91	220.5 ± 25.63	240.5 ± 33.18	260 ± 39.5
20	175 ± 21.48	201.5 ± 32.80	225 ± 38.97	244 ± 46.76	261.5 ± 54.63
50	191 ± 20.88	215.5 ± 27.90	235 ± 30.5	251 ± 34.19	270.5 ± 40.48
100	184 ± 24.68	207.5 ± 26.13	228.5 ± 31.6	236.5 ± 36.46	263.5 ± 43.99

Values are Mean ± SD.

**Table 12:** Body weight of dams during gestation period in reproductive/developmental study.

Treatment (mg/kg/d)	Day 0	1^st^ week	2^nd^ week	3^rd^ week
Control	235 ± 10.23	262.22 ± 10.92	333.33 ± 29.04	391.11 ± 8.94
20	226.0 ± 22.44	264.44 ± 18.73	314.44 ± 23.76	362.22 ± 41.52
50	223.0 ± 23.04	268.0 ± 25.98	312.77 ± 19.86	359.44 ± 26.25
100	250.6 ± 19.38	283.5 ± 19.89	320.55 ± 18.45	373.33 ± 24.63

Values are Mean ± SD of 9 animals.

**Table 13:** Absolute reproductive organ weight of male Wistar rats in reproductive/developmental study.

Treatment (mg/kg/d)	Testes	Epididymus	Seminal vesicle + prostate
Control	2.91 ± 0.41	1.20 ± 0.10	2.16 ± 0.89
20	3.21 ± 0.35	1.31 ± 0.28	2.32 ± 0.38
50	3.15 ± 0.28	1.35 ± 0.22	2.93 ± 0.70
100	3.02 ± 0.38	1.27 ± 0.19	2.80 ± 0.41

Values are Mean ± SD, n = 10.

**Table 14:** Reports of effect of 40% Garcinol on reproduction/development.

Observation Dosage (units)…	Values			
	Control	20 mg/kg/d	50 mg/kg/d	100 mg/kg/d
Pairs started (N)	10	10	10	10
Females showing evidence of copulation (N)	10	10	10	10
Females achieving pregnancy	9	9	9	9
Conceiving days 1 -5 (N)	8	9	9	9
Conceiving days 6 (N)	1	–	–	–
Pregnancy ≤21 days (N)	9	9	9	9
Pregnancy = 22 days (N)	Nil	Nil	Nil	Nil
Pregnancy ≥23 days (N)	Nil	Nil	Nil	Nil
Dams with live young born (N)	9	9	9	9
Dams with live young T day 4 PP (N)	9	9	9	9
Corpora lutea/dam (mean)	9	9	9	9
Implants/dam (mean)	11.44	9.56	10.88	11.01
Live pups/dam at birth (mean)male/Female	6.0/5.22	3.55/4.22	5.55/5.22	5.11/5.88
Live pups/dam at day 4 (mean)	6.0/5.22	3.55/4.22	5.55/5.22	5.11/5.88
Sex ratio (m/f) at birth (%)	53.46/46.54	45.71/54.29	51.55/48.45	46.46/53.54
Sex ratio (m/f) at day 4 (%)	53.46/46.54	45.71/54.29	51.55/48.45	46.46/53.54
Pup weight at birth (mean) male/Female	5.36/5.43	5.55/5.44	5.50/5.49	5.69/5.50
Pup weight at day 4 (mean) male/Female	9.66/9.77	9.86/9.81	9.69/9.65	9.85/9.69
ABNORMAL PUPS				
Dams with 0	9	9	9	9
Dams with 1	0	0	0	0
Dams with ≥ 2	0	0	0	0
LOSS OF FEMALE OFFSPRING				
Pre-implantation (corpora lutea minus implantations)	0.17	1.79	0.11	0.01
Pre-natal/post-implantations (implantations minus live births)	0.0	0	0	0
Post-natal (live births minus alive at post-natal day 4)	0	0	0	0

N-Number, PP-post partum.

## 5 Discussion

Garcinol, a compound found in the Garcinia species fruit rind is derivative of polyisoprenylated benzophenone class [5]. It has antioxidant, anticancer, anti HIV; antiulcer activities studied so far [3]. Although there are many preclinical efficacy studies being conducted on Garcinol [3,[9], [10], [11], [12], [13], [14], [15]], there are no published toxicity profiles of this molecule. We are in process of evaluating Garcinol 40% for its efficacy and thereby establishing its safety becomes of paramount importance. So we have taken up this work to study pre-clinical safety profile of standardized 40% Garcinol.

In acute single dose toxicity study, female Wistar rats were observed for clinical signs of toxicity and mortality, feed consumption, body weight gain and gross pathology and found to be normal during the study period. Further observation of animals for 14 days revealed no toxic effects. Thus, it is considered 40% Garcinol to be non-toxic at the single high dose of 2000 mg/kg.

Similarly, no abnormal clinical signs and mortality was observed in the 28 days sub acute study. Body weight gain, body temperature and feed consumption didn’t have differences in the control and 40% Garcinol treated group during the study period and in the recovery groups too. There were biologically insignificant changes in some of the haematological parameters in the few animals which were comparable to the respective controls. There was biologically insignificant decrease in creatinine and urea of female animals of mid dose groups. In the histopathological examination, few animals presented minor lesions which didn’t reveal dose response with respect to severity which shows that 40% Garcinol is non- toxic during 28 days sub acute toxicity study.

As there is potential long term use of the molecule, we established the long term safety of 40% Garcinol by taking up the 90 days sub chronic toxicity study. All the animals were observed for clinical signs, changes in body weight and feed consumption daily and were comparable among the control and treatment groups. In female animals a decrease was noticed in the WBC and MCHC levels in the high dose recovery groups and remaining parameters were unaffected. The increase in the clinical parameters was not distributed in dose related pattern and was in between physiological limits. The absolute and relative organ weight of the animals were comparable among the treated and control group. In histopathological examination, there were few spontaneous and incidental lesions which were comparable with control group. With the above observations 40% Garcinol can be characterized safe at the 90 days sub chronic study with the No Observed Adverse Effect Level of 100 mg/kg/day.

**Fig. 5:**
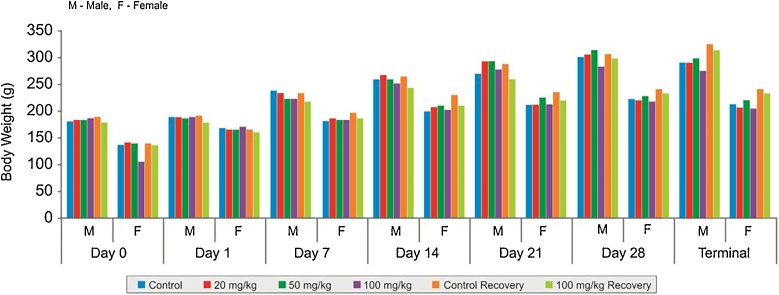
Comparison of body weights of experimental animals during the reproductive/developmental study at different time points of 40% Garcinol.

In reproductive and developmental toxicity study, there were no mortalities and clinical signs of abnormalities during the study period in any of the groups. The mean body weight of females in the gestation as well as during 4 days lactation period was comparable to control group females (Fig. 5). Further, there were no 40% Garcinol related histopathological changes in reproductive organs (testes, epididymus, ovary, uterus, seminal vesicles) of the high dose animals as well the organ weights were comparable to the control group animals. The reproductive parameters like females showing evidence of copulation, females achieving pregnancy, time taken to conceive were normal indicating lack of reproductive toxicity of 40% Garcinol. Pre- implantation and post implantation, loss at the birth and up to day 4 post partum were comparable in all groups. One female each from all the control and dose groups were non pregnant which didn’t affect the outcome of the study as per the guideline (minimum 8 pregnant animals per group). The number of live pups on the day of birth and day 4 were not changed as there was no loss of pups post partum. The male to female ratio and body weight of pups were comparable among the 40% Garcinol treated and control groups. Thus, the result obtained shows that use of standardized 40% Garcinol in the reproductive/developmental stage of the Wistar rats is safe with no observed adverse effect.

## 6 Conclusion

The 40% Garcinol did not show any adverse effect at high single dose of 2000 mg/kg at acute safety study in Wistar rats and can be classified as GHS category 5/unclassified according to the Globally Harmonized classification system (GHS) for classification of chemicals. There were no treatment related changes induced by 40% Garcinol, at highest dose of 100 mg/kg/day at 28 days repeated dose oral toxicity study, 90 days repeated dose oral toxicity and reproductive/developmental toxicity study. Thus, it can be concluded that 40% Garcinol has low toxicity profile in rodents and had no observed effects under experimental conditions applied.

## Transparency document

Transparency document
